# Caller characteristics influence recruitment to collective anti-predator events in jackdaws

**DOI:** 10.1038/s41598-018-25793-y

**Published:** 2018-05-09

**Authors:** Richard D. Woods, Michael Kings, Guillam E. McIvor, Alex Thornton

**Affiliations:** Centre for Ecology and Conservation, University of Exeter, Penryn Campus, Penryn, UK

## Abstract

Across the animal kingdom, examples abound of individuals coming together to repel external threats. When such collective actions are initiated by recruitment signals, individuals may benefit from being selective in whom they join, so the identity of the initiator may determine the magnitude of the group response. However, the role of signaller discrimination in coordinating group-level responses has yet to be tested. Here we show that in wild jackdaws, a colonial corvid species, collective responses to anti-predator recruitment calls are mediated by caller characteristics. In playbacks next to nestboxes, the calls of nestbox residents attracted most recruits, followed in turn by other colony members, non-colony members and rooks (a sympatric corvid). Playbacks in fields outside nestbox colonies, where the immediate threat to broods was lower, showed similar results, with highest recruitment to nearby colony members’ calls. Responses were further influenced by caller sex: calls from non-colony member females were less likely to elicit responsive scolding by recruits than other calls, potentially reflecting social rank associated with sex and colony membership. These results show that vocal discrimination mediates jackdaws’ collective responses and highlight the need for further research into the cognitive basis of collective actions in animal groups.

## Introduction

From army ants to human armies, groups of conspecifics across many taxa exhibit collective responses towards external threats, often in response to specific recruitment signals^1–4^. The success of defensive groups is likely to be closely linked to their size^5^, so it is important to understand the processes that determine the magnitude of collective responses. Group defences often present collective action problems, in which individuals face conflicting incentives when deciding whether to join^6,7^. In anti-predator mobbing, for example, joining a mob can provide a collective benefit by helping to drive away a potentially lethal threat^8,9^, and individuals may also benefit from gathering information about the predator^2,3^. On the other hand, approaching a predator may be highly risky, particularly for individuals in small groups, creating an incentive to defect from joining the mob and free-ride on others’ efforts^5,7,10^. Under these circumstances, the identity of the initiator may provide crucial information to individuals deciding whether to join the mobbing group, thus influencing the magnitude of the group response.

In many species, alarm vocalisations could provide an important cue to initiator identity. However, most research to date research has focused on alarm calls that elicit individual evasive behaviour rather than collective mobbing. If alarm calls signal an imminent and severe threat, failure to respond could be fatal, so individuals may benefit from responding with evasive action regardless of who produced the call. Meerkats (*Suricata suricatta*), for example, live under high predation pressure and, although their alarm calls are individually distinctive, their responses are unaffected by the identity of the caller^11^. Caller characteristics may be more likely to influence alarm responses if the level of risk is relatively low, or particular categories of individuals are especially vulnerable. Yellow-bellied marmots, *Marmota flaviventris*, for example, respond more strongly to the alarm calls of vulnerable juveniles than those of adult females, but do not appear to discriminate between the calls of different individuals within the same age/sex category^12^. Individual vocal discrimination could be advantageous if some callers are unreliable. Vervet monkeys (*Chlorocebus pygerythrus*), for instance, cease to respond the inter-group alarm calls of individuals that appear (through repeated playbacks of their calls) to “cry wolf”, calling there is no other group is present^13^. Such selective responses are not apparent, however, in the higher-risk context of anti-predator alarm calls. Here, group members habituated to a particular individual’s leopard-specific alarm calls nevertheless showed strong responses to that same individual’s eagle-specific call^13^. These results suggest that threat level plays an important role in determining the impact of individual caller identity on receivers’ responses to alarms.

Selective responses to different callers may be particularly likely if responding to a call increases rather than reduces an individual’s exposure to threats. This is the case in collective anti-predator mobbing responses, where individuals that respond to recruitment calls move *towards* the threat, placing themselves in more danger than if they did not respond^5,7^. Consequently, receivers may respond preferentially to callers with whom they are familiar^14^ or have strong social relationships. For instance, playback experiments on captive crested macaques, *Macaca nigra*, and wild dwarf mongooses, *Helogale parvula*, show that individuals responded more strongly to the recruitment calls of group members with whom they have strong social bonds^15,16^. Given that initial recruits may themselves be followed by others, and that the costs of joining a mobbing event should decline as the number of recruits increases^5^, selective responses to initiators could, in principle, have substantial effects on the magnitude of collective anti-predator responses. However, this possibility has yet to be tested.

We used playback experiments on wild jackdaws (*Corvus monedula*) to investigate whether collective responses to anti-predator recruitment calls depend on caller identity. Jackdaws breed colonially, live in structured groups with a defined linear rank hierarchy and form long-term monogamous pair bonds^17,18^. In response to threats, they produce a harsh, rattling, “scolding” call^19^, which typically serves to recruit other group members the caller’s location. Recruits may further respond with their own scolding calls, and if a predator is present the group may mob it aggressively^17,20^. Responding to a scolding call is likely to entail time and energy costs, as well as risks associated with exposure to the threat^20,21^. Jackdaws may therefore benefit from responding preferentially to scolding calls from their mate or a fellow colony member, rather than to those of unfamiliar jackdaws. Recruitment to a mate’s scolding call could have direct fitness benefits by increasing survival for both the mate and their young, and preferentially joining more familiar individuals may generate more cohesive and effective collective response^14,22^. Some authors have also suggested that collective responses may be maintained through reciprocity, with individuals assisting those that have assisted them in the past^23,24^. Given the costs of joining a mobbing event and the potential benefits of selective responses, we predicted that the number of jackdaws responding to a scolding call would depend on the identity of the caller.

We performed playbacks of scolding calls from known individuals near the nests of breeding wild jackdaws across three nestbox colonies to test whether group responses differ depending on whether the caller is (1) a member of the *resident* breeding pair at a focal nestbox, (2) a *local* bird nesting at a different nestbox within the same colony as the resident pair or (3) a *stranger* from a different colony. The calls of local rooks, *Corvus frugilegus*, a sympatric species that often breeds and forages alongside jackdaws, were used as a heterospecific controls. To test whether high levels of threat over-ride the advantages of selective responses^11^, we repeated the experiment both near to focal nestboxes, where the perceived predation threat is expected to be relatively high, and in fields away from the nestbox colonies, where the imminent threat to nesting birds is lower. We recorded the maximum number of recruits to each playback and whether recruits made scolding calls of their own. We predicted that during playbacks away from nests there would be highest responsive scolding and recruitment to playbacks of colony-members’ alarm calls, less to those of unfamiliar jackdaws from different groups, and least of all for rook calls. Given the greater threat levels, we predicted less discriminating responses to playbacks within nestbox colonies.

## Methods

### Study sites and species

All recordings and playbacks were conducted at three jackdaw colonies near Penryn in West Cornwall, UK. Colony X (50°10′22.9″N 5°07′04.1″W), is ~5 km from the other two colonies, Y (50°11′22.4″N 5°10′53.4″W) and Z (50°11′55.5″N 5°10′10.8″W) which are themselves ~1.5 km apart. The jackdaws used in the study were all free-living adults, a large proportion of which had been colour-ringed. One hundred nest boxes were spread across the three sites at naturally realistic distances from each other (5–30 m). Recording and playbacks took place during the breeding seasons of April-June 2013 and 2014. All recordings used for playbacks were from jackdaws that were individually identifiable either from their colour-ring combinations or focused behavioural monitoring to confirm ownership of a particular nestbox. The sex of each individual was determined through behavioural observations from outside the nest box and CMOS IR nest-box cameras (females are responsible for the vast majority of the incubation^18^) and later confirmed through molecular sexing^25^.

### Ethical statement

All experimental procedures and bird ringing were carried out under licenses from the Home Office (PPL 80/2371 to AT) and British Trust for Ornithology (C6079, C5752, C5746). The research was approved by the University of Exeter Biosciences Ethics committee (2014/577) and carried out in accordance with the Association for the Study of Animal Behaviour (ASAB) Guidelines for the Treatment of Animals in Behavioural Research and Teaching.

### Collecting scolding recordings

To record the scolding calls of known individuals, we approached focal nestboxes, keeping the identified bird(s) in view. In the majority cases, walking towards the nestbox within 0–10 m was sufficient to cause a scolding response. In a small number of cases, residents did not scold spontaneously, so we elicited scolding by placing a ladder against the tree/building in question and climbing to within 1 m of the nestbox. Playbacks of scolding calls recorded using these two methods are known to elicit no difference in response^26^ but to avoid any possible biases we ensured that recordings obtained using the two methods were randomly assigned to playback treatments. We recorded a minimum of ten discrete calls from each of 25 jackdaws from 23 different nest-boxes across the three sites for use in playbacks. To obtain recordings of rooks for use as controls in playbacks, we approached a rookery adjacent to jackdaw colony Y where adult rooks were nesting. All recordings were made using an Olympus LS-100 portable digital recorder, recording at 48.0 Hz/16 bit, and a Sennheiser M67/K6 directional microphone and saved as uncompressed WAV files.

### Creating playback tracks

Playback tracks were created using the software package Audacity (www.audacity.sourceforge.net). Each playback consisted of three sets of eight scolding calls spread over 15 seconds, separated by 30 second periods of silence, mimicking a natural bout of scolding calls. Tracks started and ended with 30 seconds of silence. A small proportion of the calls recorded contained audible background noise from wind or traffic, which were removed from raw recordings using the high pass filter in Audacity, filtering out only frequencies below 800 Hz which includes wind and traffic noise but does not overlap with the calls themselves. The amplitude of all playback tracks was normalised.

### Playback procedure

Playbacks were conducted using Foxpro GX7 Fury remote controlled loudspeakers. Playback volume was determined using a Voltcraft SL-100 sound level meter to calibrate the output of the speaker to the sound level recorded from a scolding jackdaw at the same distance. At each colony, playback experiments were conducted in one of two distinct locations types, labelled *Near* and *Away*, that differed in the level of threat posed by predators to nesting jackdaws and their broods. Playbacks were never conducted if the caller in the playback track could be seen in the vicinity.

During *Near* playbacks the speaker was placed directly below a focal nestbox (N = 23 different nestboxes spread across three sites), to simulate a high threat to the resident nesting birds and their chicks. We used four experimental treatments (4 treatments at 23 nestboxes = 92 playbacks). *Resident* treatments consisted of calls from one member of the pair occupying the focal nestbox, whose partner was likely to be in the vicinity, *Local* from a jackdaw of the same colony (nesting 100–300 meters of the *Resident* nestbox), *Stranger* from a jackdaw at a different colony, and *Rook* calls as a control.

The procedure for *Away* playbacks simulated a lower threat intensity, with the speaker placed in an open area 50 m from the nearest nestbox, equidistant from the focal *Resident* nest and the nest of the *Local* bird used in the *Near* playbacks (N = 23 different locations used). As the speaker was placed away from any nestbox, the distinction between *Resident* and *Local* treatments was no longer meaningful, so these treatments were combined into a single *Colony-member* treatment. After placing the speaker in position, an observer then took up a position concealed either in a car or beneath camouflaged netting with a clear view of the playback area and waited 15 minutes to allow any nearby jackdaws to return to normal behaviour. During this time the observer set up a Panasonic HC-X900 high-definition camcorder with a view encompassing the speaker at the bottom of shot and the sky for at least 50 meters in all directions above the playback location. The playback treatment (*Colony-member*, *Stranger*, or *Rook*) was then broadcast from the speaker via remote control. Treatments were conducted in random order over the period when nests contained chicks, with no more than two playbacks per day per nest-box (separated by at least four hours) to avoid habituation.

From each video, we recorded two main responses. First, we noted whether or not any jackdaws made scolding calls in response to the playback. Second, we recorded the maximum number of jackdaws recruited to the playback. Recruits were classified as any jackdaw that moved to within 30 m of the speaker (this could include circling flight, landing in a tree, or changing direction towards the source of the playback). Jackdaws that were already within 30 m of the playback area when playback commenced were only included if their behaviour changed during the playback, by scolding in response to the playback, moving towards the speaker and either landing or circling close to it. We counted the number of recruits arriving throughout the playback track, continuing until after the playback ended, until no new birds entered the frame of view and birds began to disperse. The video track was freeze-framed to enable a precise count of the total number of birds. 15% of videos were transcribed by a second coder, blind to treatments. Inter-coder reliability of the number of recruits was very high (Intraclass Correlation Coefficient^27^ = 0.97, CI = 0.92–0.99, p < 0.001).

### Acoustic distinctiveness of individual calls

To determine whether jackdaw scolding calls are individually distinctive, we extracted estimates of call duration, frequency range, fundamental frequency and the power distribution across the frequency range from 785 calls by 26 individuals of both sexes. We then conducted Principal Components Analyses to mitigate collinearity in call features, and conducted Discriminant Function Analysis (DFA) on the principal components to test the discriminability of caller identity, sex and group membership. Due to variation in the number of calls recorded from each individual, we used a permutation procedure to assess the significance of classification success. Full details of the procedure for extracting and analysing call features are given in the Supplementary Material online.

### Statistical analysis of responses to playbacks

Data were analysed using R version 3.1.1 (R Core Team, 2014). The g*lmer* function from the *lme4* package^28^ was used to run generalised linear mixed models (GLMMs) with year, batch (each three- or four-treatment set of jackdaw treatments and one rook treatment) and colony (X, Y, or Z) fitted as random terms in all models to account for repeated measures. For all models, we first performed preliminary analyses that included all treatments in order to examine the difference in response to rook and jackdaw playbacks. Following this we then analysed only jackdaw treatments so that data such as caller sex and caller identity that were unavailable for rook playbacks could be included. *Near* and *Away* data were analysed separately as they contained different treatment groups. Model simplification was carried out through stepwise deletion of non-significant terms using likelihood ratio tests to compare between models. Chi-squared and p values for each full term were obtained using the Anova function^29^. Post hoc comparisons of levels of interest within categorical variables were conducted by sequentially excluding levels from models to allow comparisons of remaining levels.

To test whether caller identity significantly affected the probability that jackdaws would produce scolding calls in response to playbacks, we fitted the presence or absence of responsive scolding (1, 0) as a binomial response term in two GLMMs, one for *Near* data and another for *Away* data. Treatment (*Resident*, *Local, Stranger*, and *Rook* for *Near* playbacks and *Colony-member, Stranger*, or *Rook* for *Away* playbacks) was fitted as an explanatory variable. Current wind speed (measured at Carnkie Weather Station, 3 km west of site Y; www.carnkieweather.co.uk) was fitted as an additional explanatory term as it could influence the attenuation of playback stimuli. When comparing jackdaw treatments, caller identity was fitted as a random term, and sex as an additional explanatory term.

The magnitude of group responses to playbacks may be influenced both by the initial playback stimulus and any subsequent responsive scolds. We therefore conducted separate analyses to examine first the effects of playback treatments on recruitment in cases where responsive scolding occurred, and second where the only scolds were produced by the loudspeaker. We conducted four GLMMs (with and without responsive scolding, both *Near* and *Away*) with the number of recruits fitted as a Poisson-distributed response. In each case, treatment and wind speed were fitted as explanatory terms, with sex fitted as an additional term for comparisons between jackdaw treatments.

## Results

### Acoustic distinctiveness of jackdaw scolding calls

Principal Components Analysis of acoustic features generated three Principal Components, each accounting for over 10% of the variance. These captured (PC1) fundamental frequency and power distribution (40.3% of variance); (PC2) the frequency range and the flatness of the power spectrum (18.9%) and (PC3) call duration (12.6%; see Supplementary material, Figs S1 and S2; Table S10). Male and female callers differed primarily in PC1, with males tending to have lower fundamental frequencies than females (Fig. S2). Using discriminant Function Analysis, the percentage of correctly classified calls was 37.8% for individual caller identity, 64.1% for sex and 54.3% for group membership. Permutation tests confirmed that identity (p < 0.001), sex (p < 0.001) and group (p < 0.001) were all significantly discriminable (Supplementary Material, Figs S2 and S3).

### Does caller identity affect the probability of responsive calling?

#### Near to nests

Treatment had a significant influence on the probability of responsive scolding for playbacks performed *Near* to nests (GLMM; χ^2^ = 12.64, d.f. = 3, *P* = 0.005). Recruits were significantly less likely to scold in response to *Rook*s than to *Locals* (χ^2^ = 23.87, d.f. = 1, *P* < 0.001), or *Strangers* (χ^2^ = 17.89, d.f. = 1, *P* < 0.001) and there was a non-significant trend for a lower probability of scolding in response to *Rooks* than *Residents* (χ^2^ = 3.61, d.f. = 1, *P* = 0.057).

**Figure 1:**
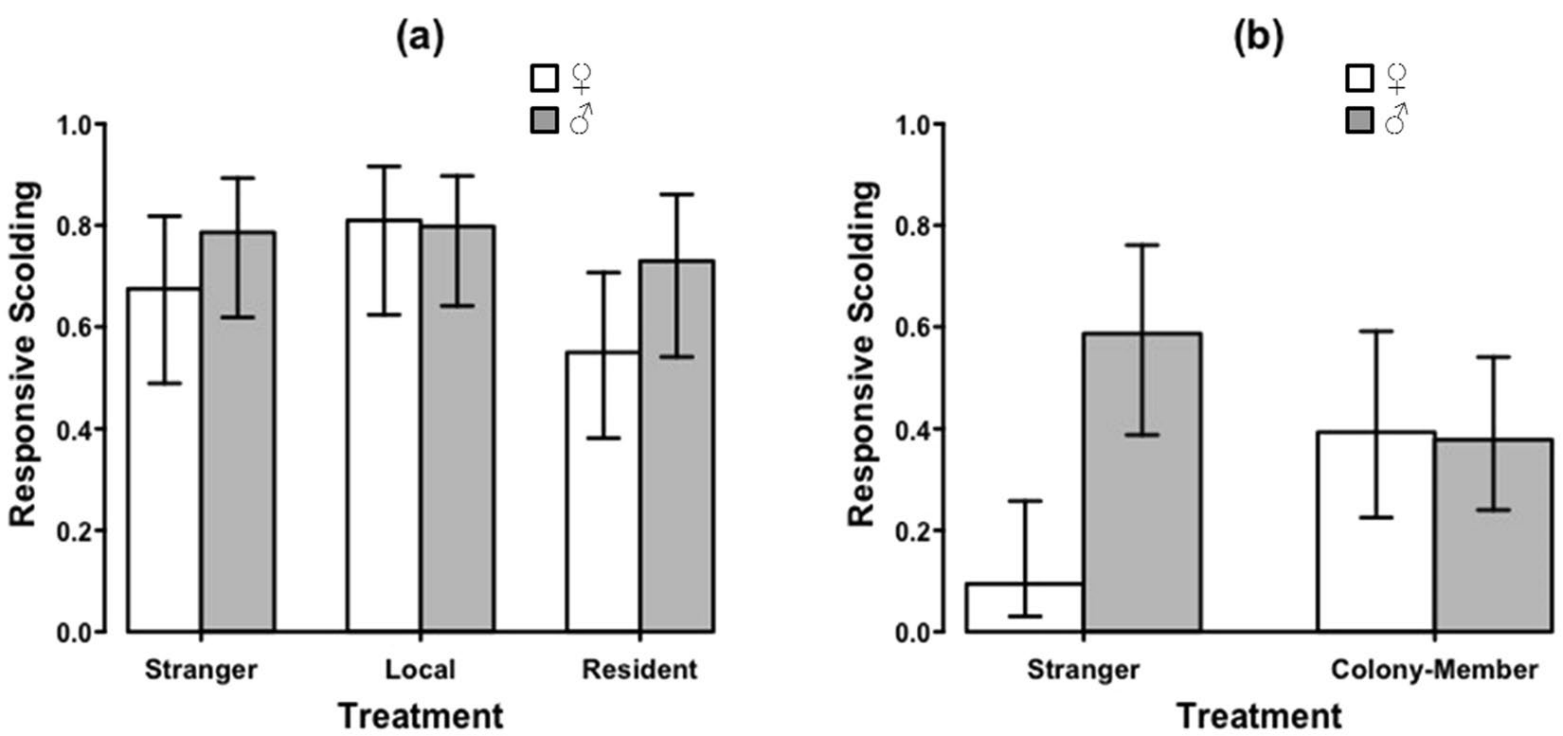
Probability of responsive scolding to jackdaw playbacks (**a**) near to and (**b**) away from nests, depending on caller sex (white = female; grey = male) and experimental treatment. Bars show means ± SE derived from minimal models.

When restricting the analysis to jackdaw treatments only (N = 66 playbacks at 23 nests; 3 playbacks were excluded from analysis as the sex of the caller was uncertain), there was no effect of treatment (GLMM; χ^2^ = 0.989, d.f. = 2, *P* = 0.610; Fig. 1a; Supplementary Table S1) or caller sex (χ^2^ = 0.686, d.f. = 1, *P* = 0.408) and there was no interaction between treatment and caller sex (χ^2^ = 0.338, d.f. = 2, *P* = 0.845).

#### Away from nests

Treatment had a significant influence on the probability of responsive scolding for playbacks performed *Away* from nests (GLMM; χ^2^ = 8.766, d.f. = 2, *P* = 0.013). Recruits were significantly less likely to scold in response to *Rooks* than jackdaw *Colony*-*members* (χ^2^ = 7.82, d.f. = 1, *P* = 0.005), or *Strangers* (χ^2^ = 5.11, d.f. = 1, *P* = 0.024).

Restricting the analysis to jackdaw treatments, there was a significant interaction between treatment and sex (GLMM; χ^2^ = 4.366, d.f. = 1, *P* = 0.037; Fig. 1b; Supplementary Table S2). Recruits were less than half as likely to scold in response to playbacks of female *Strangers* compared to male *Strangers* (χ^2^ = 6.214, d.f. = 1, *P* = 0.013), and both male (χ^2^ = 7.823, d.f. = 1, *P* = 0.005) and female *Colony-members* (χ^2^ = 5.052, d.f. = 1, *P* = 0.025).

### How does treatment affect the number of recruits?

Across all playback experiments, the number of recruits was significantly higher when jackdaws scolded in response to playbacks than when there was no responsive scolding (GLMM; est = 0.28 ± 0.07; χ^2^ = 13.21, d.f. = 1, *P* < 0.001). For ease of interpretation, we therefore analysed levels of recruitment in playbacks where responsive scolding occurred separately to cases where it did not.

#### Near to nests with responsive scolding

In cases where responsive scolding occurred following playbacks *Near* to nests, there was a significant effect of treatment on recruitment (GLMM; χ^2^ = 20.10, d.f. = 3, *P* < 0.001). Recruitment to *Rooks* was significantly higher than to *Strangers* (χ^2^ = 6.447, d.f. = 1, *P* = 0.011), but not significantly different to *Local* (χ^2^ = 2.758, d.f. = 1, *P* = 0.097), or *Resident* (χ^2^ = 0.010, d.f. = 1, *P* = 0.920) playbacks.

**Figure 2:**
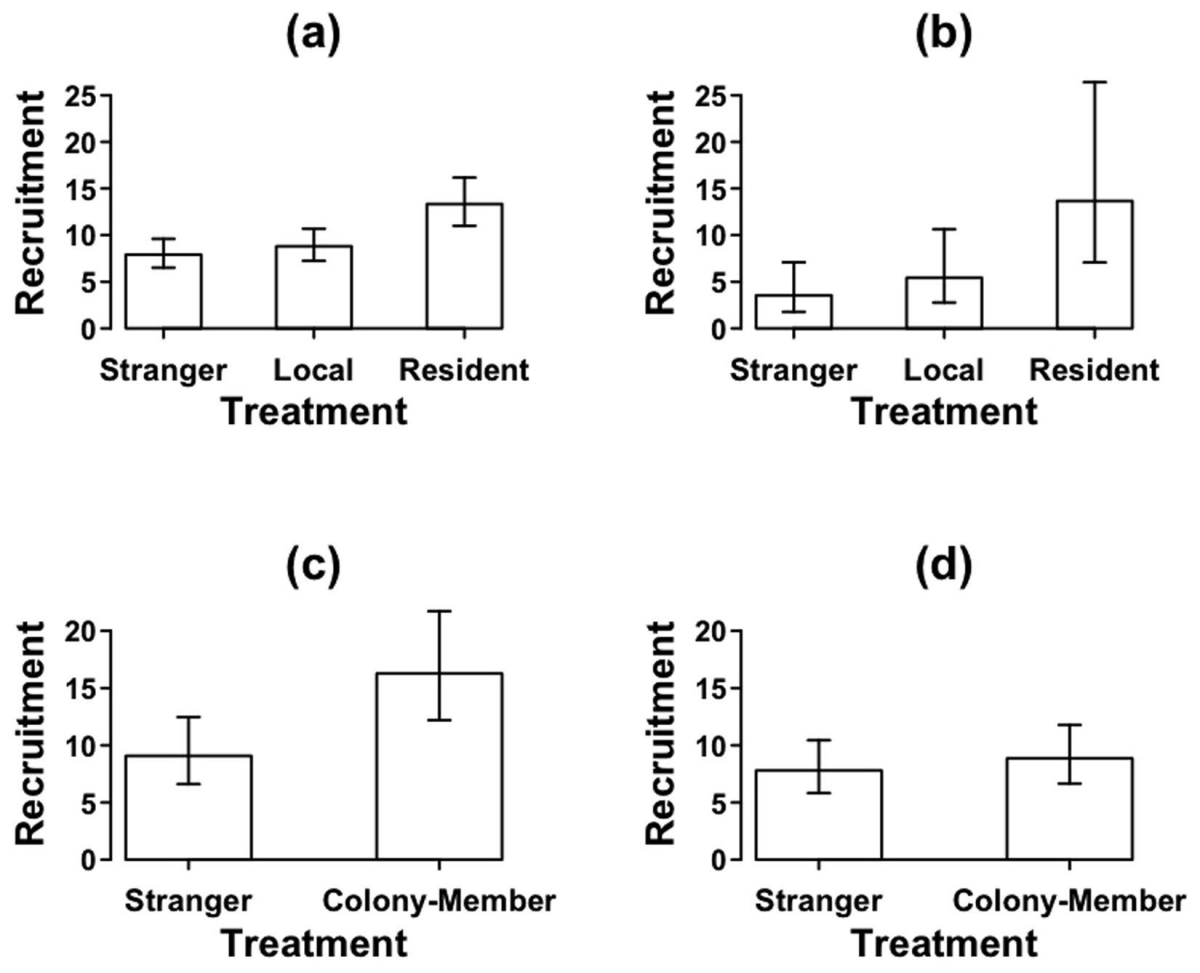
Number of jackdaws recruited to playbacks near nests when responsive scolding (**a**) occurred or (**b**) did not occur; and recruits to playbacks away from nests when responsive scolding (**c**) occurred or (**d**) did not occur. Bars show means ± SE derived from minimal models.

Restricting the analysis to jackdaw treatments only, there was a significant effect of treatment (GLMM; χ^2^ = 11.63, d.f. = 2, *P* = 0.003; Fig. 2a; Table S3). Recruitment during *Resident* playbacks was significantly higher than playbacks of *Local* (GLMM; χ^2^ = 10.21, d.f. = 1, *P* = 0.001), and *Stranger* (χ^2^ = 4.446, d.f. = 1, *P* = 0.035). Recruitment to *Local* and *Stranger* playbacks was not significantly different (χ^2^ = 1.031, d.f. = 1, *P* = 0.310). There was no significant effect of sex (χ^2^ = 0.679, d.f. = 1, *P* = 0.410), and no interaction between caller sex and treatment (χ^2^ = 1.437, d.f. = 2, *P* = 0. 488).

#### Near to nests without responsive scolding

Treatment had a significant effect on recruitment for playbacks performed *Near* to nests when no responsive scolding occurred (GLMM; χ^2^ = 66.62, d.f. = 3, *P* < 0.001). Recruitment was significantly lower for *Rook* playbacks than for any of the jackdaw treatments (*Rook* vs. *Resident:* χ^2^ = 57.91, d.f. = 1, *P* < 0.001; *Rook* vs. *Local:* χ^2^ = 12.65, d.f. = 1, *P* < 0.001; *Rook* vs. *Stranger*: χ^2^ = 7.987, d.f. = 1, *P* = 0.005).

Restricting the analysis to jackdaw treatments, there was a significant overall effect of treatment on recruitment, with the highest mean levels of recruitment in response to *Resident* calls followed by *Colony-members* and finally *Strangers* (GLMM; χ^2^ = 11.33, d.f. = 3, *P* = 0.003; Fig. 2b; Table S4). Post-hoc comparisons between treatments showed marginally non-significant trends for lower responses to *Strangers* than both *Residents* (GLMM; χ^2^ = 3.275, d.f. = 1, *P* = 0.070) and *Locals* (GLMM; χ^2^ = 3.388, d.f. = 1, *P* = 0.066), with no significant difference between *Residents* and *Locals* (GLMM; χ^2^ = 0.768, d.f. = 1, *P* = 0.380). There was no effect of sex (χ^2^ = 0.306, d.f. = 1, *P* = 0.580), and no interaction between caller sex and treatment (χ^2^ = 1.794, d.f. = 2, *P* = 0. 408).

#### Away from nests with responsive scolding

Treatment had a significant effect on recruitment for playbacks performed *Away* from nests when responsive scolding occurred (GLMM; χ^2^ = 19.85, d.f. = 2, *P* < 0.001). It was not possible to compare recruitment to Rook playbacks to that for jackdaw treatments because responsive scolding to Rook playbacks only occurred on two occasions.

Restricting the analysis to jackdaw treatments, there was a significant effect of treatment (GLMM; χ^2^ = 9.658, d.f. = 1, *P* = 0.002; Fig. 2c; Table S5) with higher recruitment in response to *Colony-member* playbacks than *Stranger* playbacks. There was no effect of sex (χ^2^ = 0.493, d.f. = 1, *P* = 0.482) and no interaction between sex and treatment (χ^2^ = 2.892, d.f. = 1, *P* = 0. 089).

#### Away from nests without responsive scolding

Treatment had a significant effect on recruitment for playbacks performed *Away* from nests when no responsive scolding occurred (GLMM; χ^2^ = 122.4, d.f. = 2, P < 0.001). Recruitment was significantly lower for *Rook* playbacks than either *Colony-members* (GLMM; χ^2^ = 116.9, d.f. = 1, P < 0.001) or *Strangers* (GLMM; χ^2^ = 5.314, d.f. = 1, P = 0.021).

Restricting the analysis to jackdaw treatments, there was no effect of treatment (GLMM; χ^2^ = 0. 690, d.f. = 1, P = 0. 406; Fig. 2d; Table S6). There was no difference in recruitment to *Colony-member*s compared to *Stranger* playbacks. There was no effect of sex (χ^2^ = 0.481, d.f. = 1, P = 0.488), and no interaction between sex and treatment (GLMM; χ^2^ = 2.259, d.f. = 1, P = 0.133).

## Discussion

A number of recent studies have shown that individual characteristics and social relationships can have substantial effects on group structure and cohesion during collective movements^30–32^. It has also long been known that, in certain species, the identity of alarm callers can affect individual receivers’ responses^12,13,15,16^. Here we provide strong evidence that alarm caller identity can also mediate the magnitude of collective responses to threats. Our analyses confirm that, like the scolding calls of other corvids^33^, jackdaw anti-predator recruitment calls are individually distinctive, and differ between the sexes. Playbacks show that jackdaw groups discriminate between different callers when responding to these calls, and that recruitment exhibits the positive feedback characteristic of collective behaviour^34^, with responsive scolding by recruits further magnifying the magnitude of the group response.

In the absence of responsive scolding, we found greater levels of recruitment in response to the calls of jackdaws than those of rooks. However, this pattern was reversed when responsive scolding by jackdaws occurred, potentially because the additive effects of calls by the two species, which associate frequently and share common predation risks^35^, magnified the intensity of the recruitment stimulus. When restricting the analyses to responses to jackdaw calls only, it is clear that it not only the species of the caller, but also its individual characteristics have important effects on group responses. When playbacks were performed *away* from nestboxes, recruits flew away from the colony towards the source of the playback. Here, the calls of *Colony-members* elicited more recruits than those of *Strangers*, but this difference occurred only when playbacks combined with the additive influence of responsive scolding by recruits. This finding is consistent with vocal discrimination on a categorical level of unfamiliar vs familiar callers^36^. However, a number of our findings suggest that jackdaws also employ more fine-scale vocal discrimination when responding to alarm calls.

First, receiver responses were affected not only by their familiarity with the caller, but also by the caller’s sex. Notably, the effects of caller sex depended on the spatial location of the playbacks, with patterns of responsive scolding to playbacks away from nests also raising the possibility that responses may be mediated by the caller’s perceived rank. Here, the responses to *Stranger* females were significantly lower than to any other treatment. There are a number of potential explanations for this result. One is that during the breeding season females tend to remain in close proximity to their nests whilst males may travel further afield in search of food for their partner and chicks^18^. Colony members may therefore be more used to hearing the calls of non-colony males than females. It is also possible that responses may be related to the caller’s rank. In jackdaw colonies, males outrank females and within the female dominance hierarchy an individual’s rank is determined by the rank of her partner^18,37^. An unknown female would therefore by default be outranked by all members of a breeding colony. If caller rank influences responses to recruitment calls, this raises the possibility that individuals could seek to signal their quality or acquire social prestige^38,39^ by responding to the calls of high ranking individuals, but work is needed to test this possibility.

Secondly, playbacks revealed important differences in responses to different individuals within a colony. When playbacks were performed *near* to nestboxes we found that recruitment increased progressively from *Stranger* to *Local* to *Resident* playbacks. This pattern was apparent when responsive scolding did not occur, but was stronger when it did. Although we ensured that we could not see the individual whose call was being played when conducting playbacks, we cannot rule out the possibility that it was in the vicinity. It is therefore possible that one member of the resident pair recognised its own call, stimulating a heightened response. However, this scenario seems unlikely because all known examples of vocal self-recognition in birds are based on syllable order rather than recognition of individual syllables^40,41^. Jackdaw scolding calls are monosyllabic so the opportunity for self-recognition of a pattern of syllables does not exist. Furthermore, as anyone who hears a recording of their own voice will appreciate, the attenuation of sound through air and the bones of the cranium means that individuals’ perception of their own voice is likely to sound different to a recording^42^.

A more plausible explanation is that the pattern of recruitment near to nests is based on discrimination between known individuals within the colony. This could be a spatial association whereby calls elicit a higher response in the location where they are most frequently heard; hence *Resident* calls played back at their own nestbox produce a stronger response. However, since birds move around and join in scolding events throughout the colony it is unlikely that a strict spatial association can account for the findings. Instead we suggest that, as suggested in studies of mammals^15,16^, social relations between caller and receiver mediate the pattern of recruitment. In our study, the rapid and dynamic movements of birds responding to playbacks made it impossible to determine the identity of individual recruits, so this conclusion must remain speculative. Nevertheless, patterns of response are consistent with a role for social relationships between callers and recruits. The nearest birds to a *Resident* playback performed at the nest are likely to be the caller’s partner and other closely associated individuals. The strength of social bond between the caller and nearby birds is likely to decrease from *Resident* to *Local* to *Stranger* and this could determine the strength of behavioural and vocal response by nearby birds which will in turn stimulate further recruitment.

In summary we show that collective anti-predator responses in jackdaws are strongly affected by caller identity, with both recruitment and responsive scolding varying between different callers. Most research on collective behaviour emphasises simple, reflexive mechanisms^34,43^, but our work demonstrates that more complex cognitive processes can play an important role in mediating collective actions. In jackdaws, the ability to discriminate between the vocalisations of different callers provides crucial information to conspecifics deciding whether to take part in costly collective events.

### Data accessibility

Data have been deposited in Figshare: doi: 10.6084/m9.figshare.5831682.

## Electronic supplementary material

Supplementary Material
