# Supplementary material for: Caller characteristics influence recruitment to collective anti-predator events in jackdaws
Source: Sci Rep. 2018 May 9;8:7343. doi: 10.1038/s41598-018-25793-y (PMC5943305; doi:10.1038/s41598-018-25793-y)
Supplement: Supplementary file 1 — Supplementary Material [file 41598_2018_25793_MOESM1_ESM.pdf]

SUPPLEMENTARY MATERIAL

Caller characteristics influence recruitment to collective anti-predator events in jackdaws

Richard D. Woods[1], Michael Kings[1], Guillam E. McIvor[1] & Alex Thornton[1*]

[1]Centre for Ecology and Conservation, University of Exeter, Penryn Campus, Penryn, UK

* Correspondence to: alex.thornton@exeter.ac.uk

This file contains:

(a) Tables of statistical results from mixed model analyses
(b) Methods and Results of acoustic analyses on call discrimination

(a) Tables of statistical results from mixed model analyses

Table S1. GLMM of factors affecting responsive scolding to jackdaw playbacks near to nests.

	**Wald statistic (χ^2)**	***df***	***P***
Wind	0.973	1	0.324
Sex	0.686	1	0.408
Treatment	0.989	2	0.610
Sex*Treatment	0.338	2	0.845

Data from 66 playbacks were fitted to a binomial distribution with binary response terms (Yes or No) indicating whether or not any scolding by recruits occurred during each playback. Playbacks were performed near to 23 nestboxes with random terms including batch (estimated variance component = 0.067, SE = 0.258), caller identity (estimated variance component = 0.603, SE = 0.777), colony (estimated variance component = 0.000, SE = 0.010), and year (estimated variance component = 0.000, SE = 0.000).

Table S2. GLMM of factors affecting responsive scolding to jackdaw playbacks away from nests.

	**Wald statistic (χ^2)**	***df***	***P***
**Full model**			
Wind	7.634	1	0.006
Sex*Treatment	4.366	1	0.037
Sex	0.320	1	0.572
Treatment	0.042	1	0.836
**Minimal Model**	**Effect size**	**SE**	
Constant	-5.030	1.760	
Wind	0.192	0.069	
Sex*Treatment	See Fig 1b		

Data from 66 playbacks were fitted to a binomial distribution with binary response terms (Yes or No) indicating whether or not any scolding by recruits occurred during each playback. Playbacks were performed at 23 locations away from nestboxes with random terms including batch (estimated variance component = 0.142, SE = 0.377), caller identity (estimated variance component = 0.000, SE = 0.000), colony (estimated variance component < 0.000, SE < 0.000), and year (estimated variance component = 0.000, SE = 0.000).

Table S3. GLMM of factors affecting recruitment near to nests when responsive scolding occurred.

	**Wald statistic (χ^2)**	***df***	***P***
**Full Model**			
Treatment	11.63	2	0.003
Sex	0.679	1	0.410
Sex*Treatment	1.437	2	0.488
Wind	0.015	1	0.901
**Minimal Model**	**Effect size**	**SE**	
Constant	2.068	0.194	
Treatment			
Stranger	0	0	
Local	0.107	0.136	
Resident	0.523	0.160	

Data from 46 playbacks were fitted to a poisson distribution with recruitment fitted as the response term indicating the number of jackdaws recruited to each playback. Playbacks were performed near to 23 nestboxes with random terms including batch (estimated variance component = 0.249, SE = 0.499), caller identity (estimated variance component = 0.084, SE = 0.289), colony (estimated variance component = 0.018, SE = 0.133), and year (estimated variance component = 0.000, SE = 0.000).

Table S4. GLMM of factors affecting recruitment near to nests when responsive scolding did not occur.

	**Wald statistic (χ^2)**	***df***	***P***
**Full Model**			
Treatment	11.33	2	0.003
Wind	6.401	1	0.011
Sex*Treatment	1.794	2	0.408
Sex	0.306	1	0.580
**Minimal Model**	**Effect size**	**SE**	
Constant	2.613	1.052	
Treatment			
Stranger	0	0	
Local	0.943	0.365	
Resident	1.099	0.308	
Wind	-0.110	0.032	

Data from 20 playbacks were fitted to a poisson distribution with recruitment fitted as the response term indicating the number of jackdaws recruited to each playback. Playbacks were performed near to 14 nestboxes with random terms including batch (estimated variance component = 0.649, SE = 0.806), caller identity (estimated variance component = 0.000, SE = 0.000), colony (estimated variance component = 0.000, SE = 0.000), and year (estimated variance component = 1.757, SE = 1.326).

Table S5. GLMM of factors affecting recruitment away from nests when responsive scolding occurred.

	Wald statistic (χ^2)	*df*	*P*
**Full Model**			
Treatment	9. 658	1	0.002
Sex*Treatment	2.892	1	0.089
Sex	0.493	1	0.482
Wind	0.100	1	0.752
**Minimal Model**	**Effect size**	**SE**	
Constant	2.179	0.303	
Treatment			
Stranger	0	0	
Colony-member	0.580	0.168	

Data from 24 playbacks were fitted to a Poisson distribution with recruitment fitted as the response term indicating the number of jackdaws recruited to each playback. Playbacks were performed at 24 locations away from nestboxes with random terms including batch (estimated variance component = 0.915, SE = 0.957), caller identity (estimated variance component = 0.087, SE = 0.296), colony (estimated variance component = 0.000, SE = 0.000), and year (estimated variance component = 0.000, SE = 0.000).

Table S6. GLMM of factors affecting recruitment away from nests when responsive scolding did not occur.

	**Wald statistic (χ^2)**	***df***	***P***
Sex*Treatment	2.259	1	0.133
Wind	1.165	1	0.280
Treatment	0.690	1	0.406
Sex	0.481	1	0.488

Data from 42 playbacks were fitted to a Poisson distribution with recruitment fitted as the response term indicating the number of jackdaws recruited to each playback. Playbacks were performed at 20 locations away from away from nestboxes with random terms including caller identity (estimated variance component = 2.309, SE = 1.519), colony (estimated variance component = 0.171, SE = 0.413 and year (estimated variance component = 0.000, SE = 0.000). Batch was not included as it caused the model to fail to converge.

(b) Methods and Results of acoustic analyses on call discrimination

- **Call feature extraction**

We analysed the "scolding" alarm calls of male and female jackdaws from each of three different colonies were recorded during the 2015 breeding season (26 individuals, 785 calls). The number of calls recorded for each individual varied (Table S7). Prior to call feature extraction, a high-pass filter was applied to all calls to remove background noise. The high-pass filter was created using the MATLAB signal processing toolbox (MATLAB 2014a; Finite Impulse Response (FIR) filter; frequency: pass band = 600Hz, stop band = 500Hz; amplitude: at pass band = 65dB, at stop band = 0.5dB). After filtering, calls that still contained excessive noise were removed from the dataset.

Estimates of call duration, frequency range, fundamental frequency and the power distribution across the frequency range were calculated for each call. Measures of call

duration, frequency range and energy distribution were calculated using quantiles of overall call energy, whereby call energy was calculated as the sum of power spectral density (PSD) across the entire duration and frequency range of a call (Charif *et al*., 2010). Call duration was measured as the difference between the times at which 5% and 95% of call energy occurred (Charif *et al*., 2010; Yorzinski *et al.*, 2006); the distribution of call energy over the duration of the call was calculated from the cumulative distribution of PSD estimates from the call spectrogram (1024-point Fast Fourier Transform (FFT), hamming window, 90% overlap). Frequency range was measured as the difference between the frequencies at which 5% and 95% of call energy occurred (Charif *et al*., 2010; Yorzinski *et al.*, 2006), as calculated from the power spectrum of the call (MATLAB Periodogram function, Hamming window, 1024-point FFT). Additionally, the frequencies below which 25%, 50% and 75% of call energy occurred were recorded (Charif *et al*., 2010; Yorzinski *et al.*, 2006). Wiener entropy (e.g. Tchernichovski et al., 2000) is a measure of the evenness of the power distribution across the frequency range and was calculated as the ratio of the geometric and arithmetic means of the PSD values of the power spectrum. The peak of the real cepstrum and an auto-correlation method (de Cheveigné & Kawahara, 2002) were used to obtain fundamental frequency estimates of calls; to reduce the number of spurious estimates generated due to noise, fundamental frequency estimation was restricted within the robust frequency range (i.e. 5-95% call energy).

Jackdaw alarm calls were typically half a second in duration (Table S8). Low Wiener Entropy indicates that there was typically a well-defined harmonic structure to the calls (Table S8). The majority of call energy was concentrated in the 1000-3000 Hz region of the frequency range, which contains the region in which the fundamental frequency estimates were typically located (Table S8).

- **Analysis and Results**

To mitigate colinearity in call features, principal component analysis was performed prior to statistical analysis. Three principal components exceeded the Kaiser-Guttman selection criterion (Kaiser, 1991) and described 71.8% of the total variance in the call features (Table S9). The three components explained 40.3%, 18.9% and 12.6% of variance respectively (Table S9). The first principal component comprises the fundamental frequency estimates and measures of power distribution across the frequency range (Table S10); the second

component describes the extent of the frequency range and the flatness of the power spectrum and the third component explains call duration (Figure S1). Male and female callers differed primarily in the first principal component (Figure S2). Hence, male callers tended to have lower fundamental frequencies than females. There was negligible sex difference in frequency range/spectral flatness and call duration (Figure S2).

Discriminant function analysis (DFA) was conducted on the principal components to test the discriminability of caller identity, sex and group membership. Due to sampling variance (Table S7), a permutation procedure (Mundry & Sommer, 2007) was required to assess significance of classification success. DFA with leave-one-out cross validation was applied to the un-permuted dataset in R (R Core Team, 2017) using the "lda" function from the "MASS" package (Ripley *et al.*, 2013) and the percentage of correctly classified calls was recorded for caller identity (37.8%), sex (64.1%) and group membership (54.3%). A thousand permuted datasets were created by randomization of caller identity, sex or group labels and classification success was recorded for each permuted dataset. Sex labels were randomized within groups and group labels were randomized within sex (see Mundry & Sommer, 2007) to control for the possible influence of between-group variation on sex discrimination and *vice versa*. The significance of classification success was determined by dividing the number of permuted datasets with classification success greater than the un-permuted dataset by the total number of datasets generated (Figure S3). Caller identity ($p<0.001$), sex ($p<0.001$) and group ($p<0.001$) were all significantly discriminable.

Table S7: Summary of composition of dataset

Individual	Sex	Group	Number of Calls
1	M	X	12
2	M	X	162
3	F	X	1
4	F	X	36
5	M	X	9
6	F	X	21
7	F	X	4
8	M	X	11
9	F	X	14
10	F	Y	11
11	M	Y	16
12	F	Y	37
13	M	Y	16
14	F	Y	24
15	M	Y	40
16	F	Y	32
17	F	Z	33
18	F	Z	38
19	M	Z	40
20	F	Z	37
21	M	Z	11
22	M	Z	109
23	F	Z	22
24	M	Z	14
25	F	Z	25
26	F	Z	10

Table S8: Descriptive statistics for call features.

Acoustic Feature	Minimum	Median	Maximum	IQR
25% Frequency (Hz)	904	1593	2326	258
50% Frequency (Hz)	1292	1938	4522	345
75% Frequency (Hz)	1507	2283	5168	301
Wiener Entropy (Au)	0.00015	0.00284	0.0524	0.00195
Cepstrum Peak (Hz)	678	1378	2756	278
Duration (s)	0.194	0.462	0.864	0.147
Frequency Range (Hz)	904	3445	6848	1464
YIN Fundamental Frequency (Hz)	558	1743	3634	608

Table S9: PCA summary

Acoustic Feature	PC1	PC2	PC3
Eigenvalue	1.7946	1.229853	1.00409
Proportion of Variance	0.40257	0.18907	0.12602
Cumulative Proportion	0.40257	0.59164	0.71767

Table S10: PCA Loadings

Acoustic Feature	PC1	PC2	PC3
25% Frequency	0.474837	-0.2047	0.01082
50% Frequency	0.493456	-0.0287	0.020262
75% Frequency	0.412234	0.283604	0.06904
Wiener Entropy	0.164913	0.602672	0.071909
Cepstrum Peak	0.344731	-0.38427	-0.0598
Duration	-0.02072	0.061068	-0.98542
Frequency Range	0.241504	0.54327	-0.08429
YIN Fundamental Frequency	0.395353	-0.25903	-0.08829

FIGURES

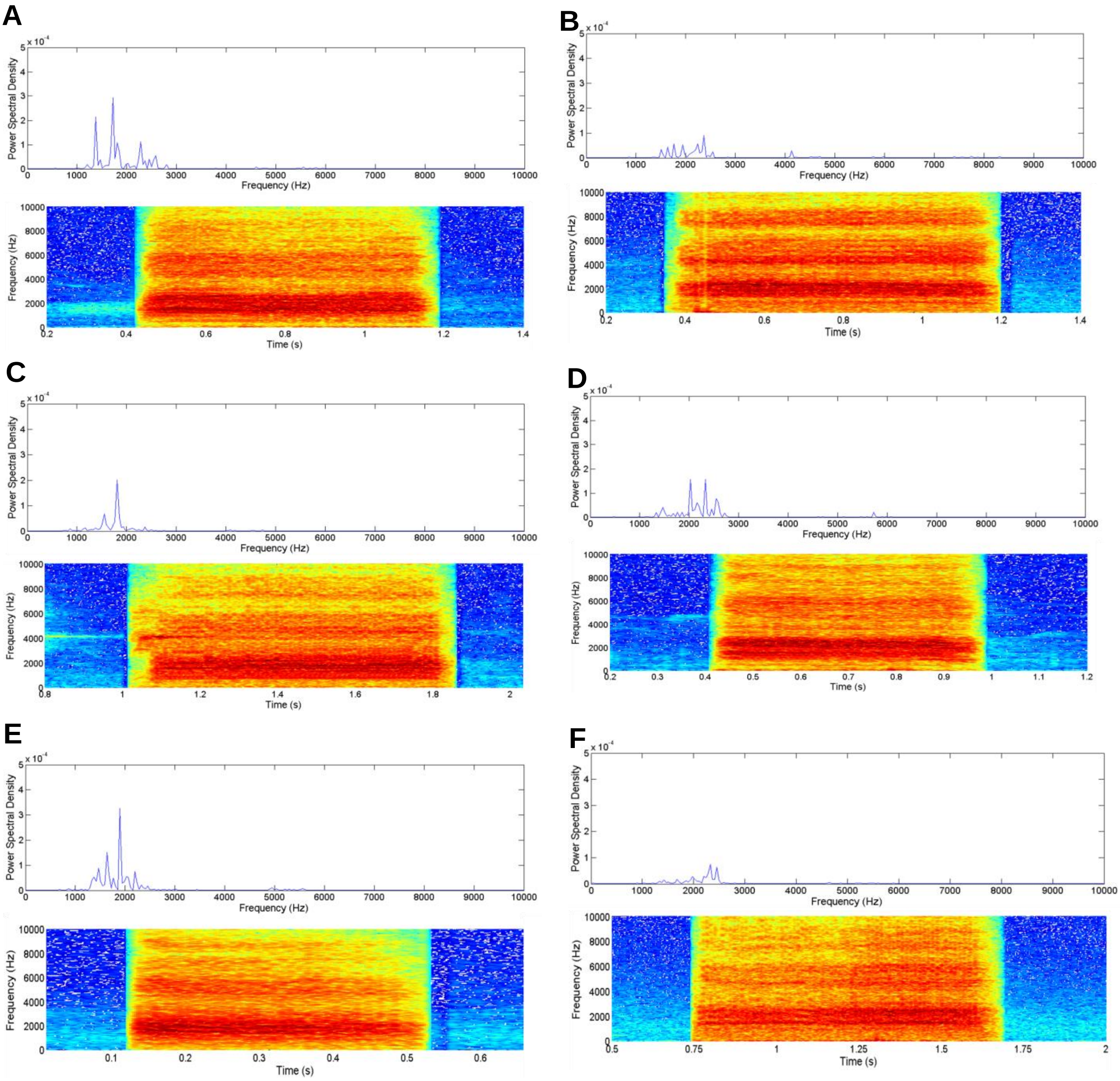

Figure S1: Power spectrum and spectrogram for the highest and lowest principal component scores for PC1 (High: **A**, Low: **B**), PC2 (High: **C**, Low: **D**) and PC3 (High: **E**, Low: **F**).

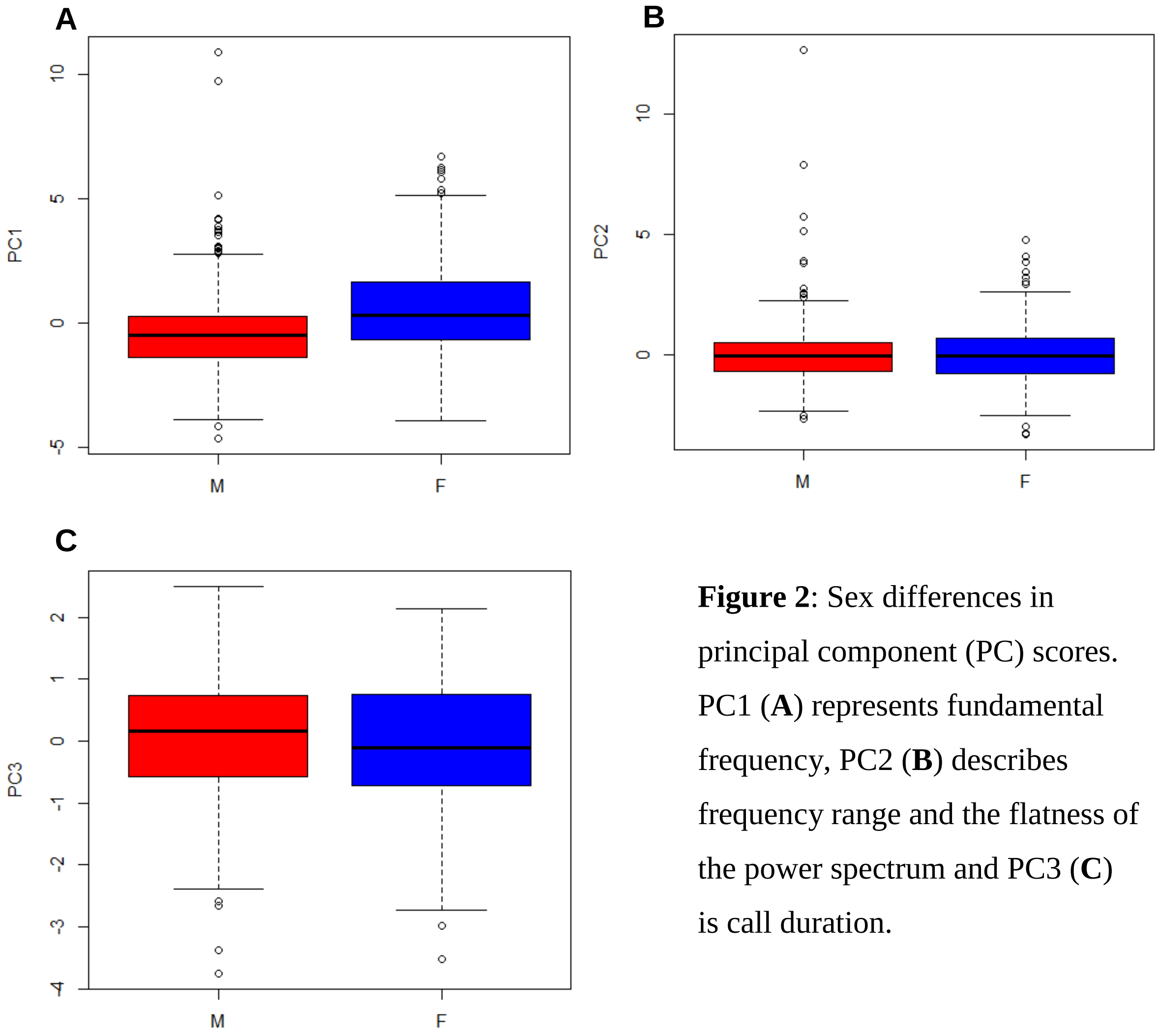

Figure 2: Sex differences in principal component (PC) scores. PC1 (**A**) represents fundamental frequency, PC2 (**B**) describes frequency range and the flatness of the power spectrum and PC3 (**C**) is call duration.

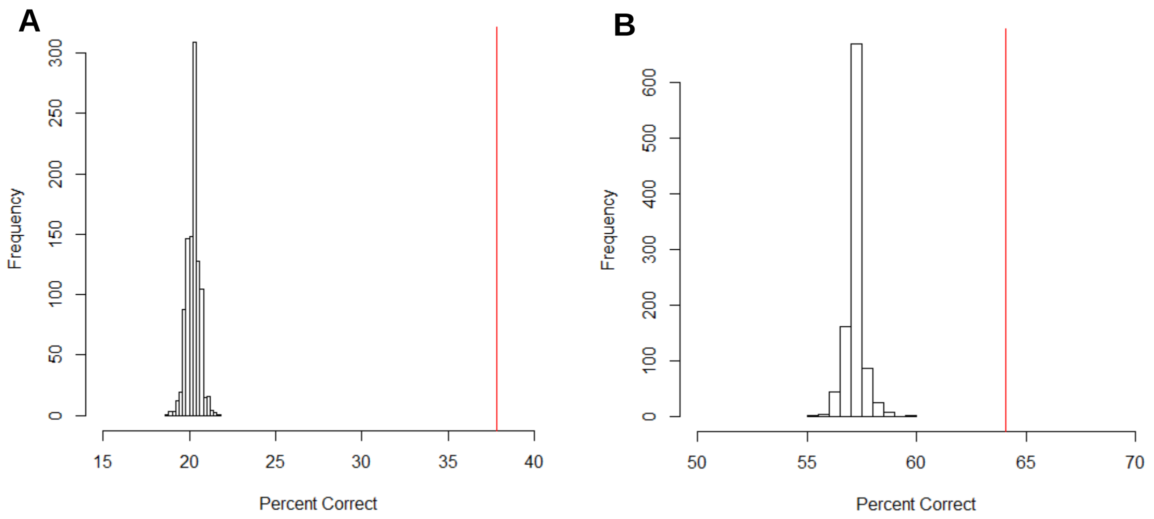

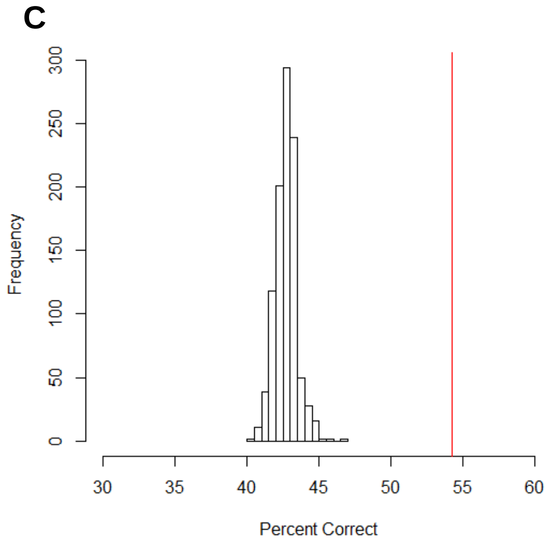

Figure 3: Histogram of classification success from Discriminant Function Analysis conducted on 1000 permuted datasets and the classification success for the un-permuted dataset (red reference line) for classification of caller identity (**A**), sex (**B**) and group membership (**C**).

REFERENCES

Charif, R.A., Waack, A.M. & Strickman, L.M. 2010 *Raven Pro 1.4 User's Manual*. Cornell Lab of Ornithology, Ithaca, NY.

de Cheveigné, A. & Kawahara, H. 2002 YIN, a fundamental frequency estimator for speech and music. *The Journal of the Acoustical Society of America* **111**: 1917-1930.

Kaiser, H.F. 1991 Coefficient Alpha for a Principal Component and the Kaiser-Guttman Rule. *Psychological Reports* **68** 855-858.

MATLAB Release 2014a, The MathWorks, Inc., Natick, Massachusetts, United States.

Mundry, R. & Sommer, C. 2007 Discriminant function analysis with nonindependent data: consequences and an alternative. *Anim. Behav.* **74**: 965-976.

R Core Team (2017). R: A language and environment for statistical computing. R Foundation for Statistical Computing, Vienna, Austria. https://www.R-project.org/.

Ripley, B., Venables, B., Hornik, K., Gebhardt, A., Firth, D. 2013. Package "MASS". Available at: cran.r-project.org/web/packages/MASS

Tchernichovski, O., Nottebohm, F., Ho, C.E., Bijan, P., Mitra, P.P. (2000). A procedure for an automated measurement of song similarity. *Anim. Behav.* **59**, 1167-1176.

Yorzinski, J.L., Vehrencamp, S.L., Clark, A.B. & McGowan, K.J. 2006 The inflected alarm caw of the American crow: differences in acoustic structure among individuals and sexes. *The Condor* **108**: 518-529.
